# Simultaneous Classical Communication and Quantum Key Distribution Based on Plug-and-Play Configuration with an Optical Amplifier

**DOI:** 10.3390/e21040333

**Published:** 2019-03-27

**Authors:** Xiaodong Wu, Yijun Wang, Qin Liao, Hai Zhong, Ying Guo

**Affiliations:** 1 School of Automation, Central South University, Changsha 410083, China; 2 Jiangsu Key Construction Laboratory of IoT Application Technology, Wuxi Taihu University, Wuxi 214064, China

**Keywords:** simultaneous, classical communication, quantum key distribution, plug-and-play configuration, optical amplifier

## Abstract

We propose a simultaneous classical communication and quantum key distribution (SCCQ) protocol based on plug-and-play configuration with an optical amplifier. Such a protocol could be attractive in practice since the single plug-and-play system is taken advantage of for multiple purposes. The plug-and-play scheme waives the necessity of using two independent frequency-locked laser sources to perform coherent detection, thus the phase noise existing in our protocol is small which can be tolerated by the SCCQ protocol. To further improve its capabilities, we place an optical amplifier inside Alice’s apparatus. Simulation results show that the modified protocol can well improve the secret key rate compared with the original protocol whether in asymptotic limit or finite-size regime.

## 1. Introduction

Quantum key distribution (QKD) is one of the most active areas in quantum information science, which promises to generate a secure key between two authenticated parties (Alice and Bob) over insecure quantum and classical channels [1,2,3,4]. The security of a key is guaranteed by the fundamental laws of quantum mechanics [5,6]. Generally speaking, there are two main approaches for the implementation of QKD, namely, discrete-variable (DV) QKD [7] and continuous-variable (CV) QKD [8,9,10,11]. Different from the DVQKD, in CVQKD, there is no requirement to use expensive single-photon detectors. Instead, the key bits are encoded in the quadrature variables (*X* and *P*) of the optical field, and the secret key bits are decoded through high-efficiency homodyne or heterodyne detection techniques [12,13,14,15].

At present, the CVQKD protocol, especially for the Gaussian-modulated coherent-state (GMCS) scheme, has been demonstrated over 100-km telecom fiber through controlling excess noise [16] and designing high-efficiency error correction codes [17,18,19]. From a practical point of view, the hardware needed in the implementation of the GMCS QKD is amazingly similar to that needed in classical coherent optical communication [20]. On the basis of this similarity, it is viable to take advantage of the same communication facility for both QKD and classical communication.

Recently, a simultaneous classical communication and quantum key distribution (SCCQ) scheme was proposed [21,22]. In this scheme, the Gaussian distributed random numbers for GMCS QKD and bits for classical communication are encoded on the same weak coherent pulse and decoded through the same coherent receiver, which provides a more cost-effective solution in practice. However, a major obstacle to the SCCQ scheme is that it can only tolerate a very small amount of phase noise [21]. This problem could lead to poor performance and thus obstruct its further development.

In this paper, we propose a SCCQ protocol based on plug-and-play configuration with an optical amplifier. Different from the GMCS QKD protocols using a true local oscillator (LO) [23,24,25], the plug-and-play CVQKD scheme waives the necessity of using two independent frequency-locked laser sources and automatically compensating the polarization drifts [26]. Since the LO and signal pulses are generated from the same laser source, we can obtain a small phase noise which can be tolerated by the SCCQ protocol in the framework of plug-and-play configuration. To further improve its capabilities, we insert an optical amplifier (OA) at the output of the quantum channel [27,28,29]. The modified protocol (SCCQ protocol based on plug-and-play configuration with an OA) can well increase the secret key rate by compensating the imperfection of Alice’s detector with only little cost in transmission distance. Here, both the asymptotic limit and the finite-size regime are taken into consideration.

This paper is structured as follows. In Section 2, we first introduce the plug-and-play dual-phase modulated coherent states (DPMCS) scheme, then present the model of SCCQ protocol based on plug-and-play configuration and the proposed modified protocol. In Section 3, we perform the noise analysis and show numeric simulations in view of practical system parameters. Finally, conclusions are drawn in Section 4. Detailed calculation of equations is shown in the Appendix.

## 2. Protocol Description

In this section, we first introduce the plug-and-play DPMCS protocol. Then, we present the model of SCCQ protocol based on the plug-and-play configuration and its modified protocol (with OA). To simplify the analysis, we adopt the binary phase-shift keying (BPSK) modulation for the classical communication and the GMCS for QKD protocol in this paper.

### 2.1. The Plug-and-Play DPMCS Protocol

**Figure 1:**
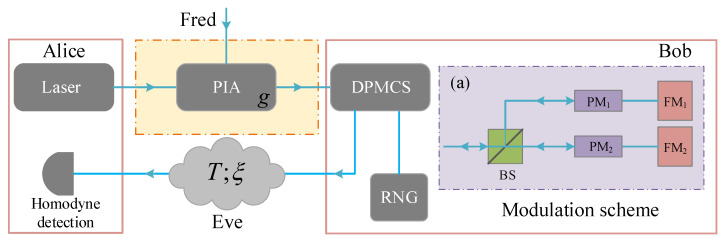
The prepared-and-measure scheme of plug-and-play dual-phase modulated coherent states (DPMCS) protocol. (**a**) Gaussian modulation scheme by using two phase modulators. PIA, phase insensitive amplifier; RNG, random number generator; PM, phase modulator; FM, Faraday mirror.

The prepare-and-measurement scheme of plug-and-play DPMCS protocol is illustrated in Figure 1. The source of light is sent from Alice to Bob, then Bob performs the dual-phase-modulation work after receiving the light. During the modulated process, random numbers drawn from a random number generator are utilized to modulate the amplitude and phase quadrature (X and P quadrature). This is really different from previous one-way CV-QKD protocols where the symmetrical Gaussian modulation is performed at Alice’s side. When Bob completes the modulation work, the dual-quadrature modulated coherent-state is directly reflected to Alice with the help of Faraday mirrors. After passing through the untrusted channel characterized by transmittance *T* and excess noise ξ, Alice receives the modulated signal. Then, she performs homodyne detection to measure the incoming mode. After this, Alice can obtain the list of data which is correlated with the list of Bob. Note that this correction is important in generating a secret key through error reconciliation and privacy amplification. Here, the classical source mentioned above is controlled by Fred [26]. Besides, the untrusted source noise is characterized by taking advantage of a phase-insensitive amplifier (PIA) with a gain of *g*. In such a practical scheme, the detector used by Alice features an electronic noise $\upsilon_{el}$ and an efficiency η. Therefore, the detector-added noise referred to Alice’s input can be expressed as $\chi_{hom}=\left[\left(1-\eta \right)+\upsilon_{el}\right]/\eta$.

### 2.2. SCCQ Protocol Based on Plug-and-Play Configuration

**Figure 2:**
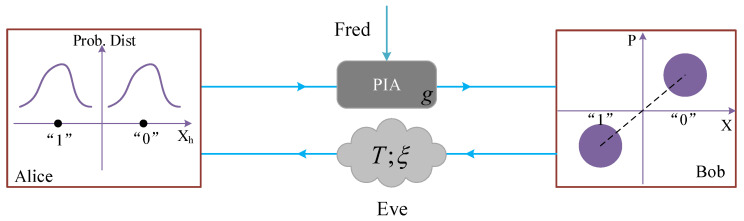
Simultaneous classical communication and quantum key distribution (SCCQ) protocol based on plug-and-play configuration. The probability distributions of X-quadrature measurement is shown at Alice’s side.

In the BPSK modulation scheme, the bit value $k_{B}$ is encoded by $|e^{-ik_{B}\pi }\alpha \rangle$, where α is a real number. While, in plug-and-play DPMCS protocol, Bob prepares coherent state $|x_{B}+ip_{B}\rangle$ and transmits it to Alice. Here $x_{B}$ and $p_{B}$ are assumed to be Gaussian random numbers with zero mean and a variance of $V_{B}N_{0}$, where $N_{0}$ represents the shot-noise variance. The SCCQ protocol based on plug-and-play configuration is straightforward and combines these two communication schemes. Namely, as shown in Figure 2, both the classical bit $k_{B}$ and Gaussian random numbers $\left\{x_{B},p_{B}\right\}$ are encoded on a coherent state $|\left(x_{B}+e^{-ik_{B}\pi }\alpha \right)+i\left(p_{B}+e^{-ik_{B}\pi }\alpha \right)\rangle$. It is remarkable that in the plug-and-play DPMCS protocol, Alice performs homodyne detection to measure either the *X* or *P* quadrature of each incoming signal. In order to obtain deterministic classical communication, the same classical bit $k_{B}$ should be encoded on both *X* and *P* quadratures.

Suppose Alice measures the *X* quadrature (*P* quadrature) and obtains the measurement result $x_{h}$ ($p_{h}$). The sign of $x_{h}$ ($p_{h}$) can be utilized to determine a classical bit $k_{B}$. In other words, the value of $k_{B}$ is assigned as 0 if $x_{h}$ ($p_{h}$) > 0 and the value of $k_{B}$ is assigned as 1 if $x_{h}$ ($p_{h}$) < 0. Note that according to the overall transmittance Tη and the value of $k_{B}$, Alice’s measurement result can be rescaled and displaced to generate a secure key, which is given by

(1) $$\begin{array}{c} \begin{array}{cc} x_{A} & =\frac{x_{h}}{\sqrt{T\eta }}+\left(2k_{B}-1\right)\alpha ,\\ p_{A} & =\frac{p_{h}}{\sqrt{T\eta }}+\left(2k_{B}-1\right)\alpha . \end{array} \end{array}$$

On the basis of the raw keys $\left\{x_{B},x_{A}\right\}$ and $\left\{p_{B},p_{A}\right\}$, Alice and Bob can distill a secure key by proceeding with classical data postprocessing, as in the case of traditional GMCS QKD.

The prepare-and-measurement (PM) version of our protocol shown above is equivalent to the entanglement-based (EB) version. In the EB scheme, Fred prepares a three-mode entanglement state $|\Phi_{ABF}\rangle$. Bob keeps one mode (*B*) with variance $V=V_{B}+1$ and measures it by using a heterodyne detector. The other mode ($A_{0}$) is sent to Alice through an untrusted quantum channel. At Alice’s side, a beam splitter with transmission η is taken advantage of to model her detector inefficiency, while an EPR state of variance $\upsilon_{el}$ is utilized to model its electronics. For the homodyne detection case, we have $\upsilon_{el}=\eta \chi_{hom}/\left(1-\eta \right)=1+\upsilon_{el}/\left(1-\eta \right)$. Finally, to distill the secret key, Alice and Bob perform information reconciliation and privacy amplification procedures. Here, we mainly consider the reverse reconciliation since it has been proved to provide a great advantage in performance of QKD schemes [11].

### 2.3. Addition of an Optical Amplifier

In practice, becuase of some inherent imperfection inevitably existing in Alice’s detection apparatus, the ideal detection process cannot be achieved. Therefore, we can only obtain a lower secret key rate than expected. In order to improve the performance of our protocol, here, an optical amplifier is applied to compensate for the detectors’ imperfections. In the following, two types of amplifiers are considered, namely, the phase-sensitive amplifier (PSA) and phase-insensitive amplifier (PIA).

*Phase-sensitive amplifier*. The PSA can be deemed as a degenerate amplifier which allows ideally noiseless amplification of a chosen quadrature. We use a matrix $\Xi^{PSA}$ to describe its properties, which is given by

(2) $$\Xi^{PSA}=\left(\begin{array}{cc} \sqrt{G} & 0\\ 0 & \sqrt{\frac{1}{G}} \end{array}\right),$$

where *G* represents the gain of amplification and G≥1.

*Phase-insensitive amplifier*. The PIA can be regarded as a non-degenerate amplifier, which is able to amplify both quadratures symmetrically. Different from the PSA, the amplification process of the PIA is related to the inherent noise. The transform of the PIA can be modeled as

(3) $$\Xi^{PIA}=\left(\begin{array}{cc} \sqrt{g}I_{2} & \sqrt{g-1}\sigma_{z}\\ \sqrt{g-1}\sigma_{z} & \sqrt{g}I_{2} \end{array}\right).$$

The inherent noise of the PIA can be given by

(4) $$\Xi_{noise}=\left(\begin{array}{cc} N_{s}I_{2} & \sqrt{N_{s}^{2}-1}\sigma_{z}\\ \sqrt{N_{s}^{2}-1}\sigma_{z} & N_{s}I_{2} \end{array}\right),$$

where *g* is the gain of the PIA and $N_{s}$ stands for variance of noise. We have introduced the PIA in the above analysis. Different from the PIA which is inserted into the output of the quantum channel in our protocol, the PIA is placed at the channel to characterize the untrusted source noise. That is to say the gain *g* of the PIA can be used to weight the source noise in the plug-and-play scheme.

**Figure 3:**
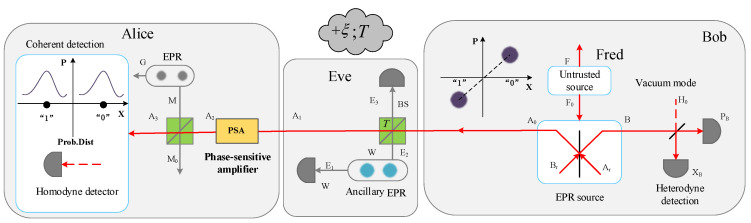
Schematic diagram of the modified protocol (SCCQ protocol based on plug-and-play configuration with an optical amplifier).

As illustrated in Figure 3, after the amplification process, mode $A_{3}$ is measured using Alice’s detector. A beam splitter with transmission η is taken advantage of to model her detector inefficiency. Besides, an EPR state of variance $\upsilon_{el}$ is utilized to model its electronics. It is worth mentioning that we adopt homodyne detection in our scheme, thus it is suitable for us to choose the PSA to compensate for Alice’s apparatus imperfection [27,28]. Then, the modified parameter $\chi_{hom}^{PSA}$ for this case is given by

(5) $$\chi_{hom}^{PSA}=\frac{\left(1-\eta \right)+\upsilon_{el}}{G\eta }.$$

Consequently, we can achieve the modified secure key rate $\hat{K}$ by substituting $\chi_{hom}^{PSA}$ for $\chi_{hom}$ in homodyne detection case.

## 3. Performance Analysis and Discussion

The noises which originated from the practical system have important effects on the performance of the SCCQ protocol. In this section, we first introduce the noise model which we adopt in this paper and present the computation of the BER in BPSK modulation scheme. Then, we show and discuss the simulation results.

### 3.1. Noise Model of SCCQ Protocol Based on Plug-and-Play Configuration

Note that the main noise sources analyzed here are (1) the detector noise assumed as $\upsilon_{el}$, (2) the vacuum noise, (3) the excess noise due to the untrusted sources denoted by $\zeta_{s}$, (4) excess noise $\xi_{RB}$ caused by Rayleigh backscattering photons, (5) the Gaussian modulation for QKD with a variance of $V_{B}$. All the noises mentioned above are defined in the shot-noise unit.

Now let’s calculate the BER of the BPSK modulation scheme, which is expressed by [21]

(6) $$BER=\frac{1}{2}erfc\left(\frac{\sqrt{T\eta \alpha }}{\sqrt{2N_{0}\left(V_{B}T\eta +\upsilon_{el}+1\right)}}\right),$$

where erfc(x) represents the complementary error function. In order to make the value of BER small enough in the classical channel, namely, obtain a BER of $10^{-9}$, the displacement α is required as

(7) $$\alpha =\frac{4.24\sqrt{V_{B}T\eta +\upsilon_{el}+1}}{\sqrt{2T\eta }}.$$

**Figure 4:**
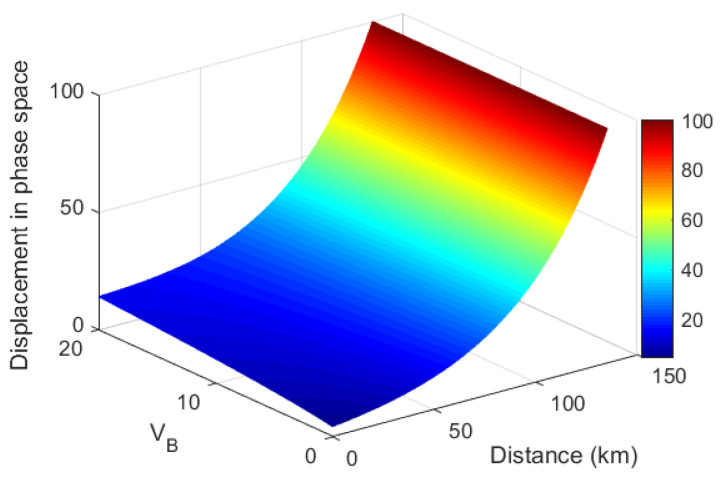
The required displacement α as a function of modulation variance $V_{B}$ and transmission distance to obtain a BER of $10^{-9}$ in the classical channel. Parameters γ=0.2dB/km, η=0.5, and $\upsilon_{el}=0.1$.

The numerical simulations of the required displacement α as a function of the transmission distance and modulation variance $V_{B}$ are illustrated in Figure 4. It shows that the longer transmission distance needs a larger displacement α for a typical modulation variance $V_{B}$ in the range of 1 to 20.

The untrusted source noise $\zeta_{s}$ is deemed to be one of the most important excess noises in the plug-and-play configuration. It can be expressed as $\zeta_{s}=\left(g-1\right)+\left(g-1\right)V_{I}$, where *g* is a gain of a PIA and $V_{I}$ is the noise variance of a vacuum state $\left(X_{I},P_{I}\right)$. That is to say, the untrusted source noise $\zeta_{s}$ can be weighted by parameter *g*.

The other excess noise we need to consider here is $\xi_{RB}$, which is caused by Rayleigh backscattering photons. Since the reflected light in the plug-and-play configuration is of the same frequency as the initial laser source, we cannot use the “in-band” photon to filter or attenuate it. The excess noise $\zeta_{RB}$ is given by

(8) $$\zeta_{RB}=\frac{2\langle N_{RB}\rangle }{\eta T},$$

where $\langle N_{RB}\rangle$ is Rayleigh backscattering photons. Then, the backscattered photons $\langle N_{RB}\rangle$ per second $\Delta_{B}$ is expressed as [30]

(9) $$\Delta_{B}=\frac{\varpi \left(1-10^{-2\gamma L/10}\right)V_{B}R}{2\eta_{B}T},$$

where ϖ stands for the the Rayleigh backscattering coefficient, $\eta_{B}$ represents the insertion loss inside Bob (round-trip), *R* represents the system repetition rate, γ is a fiber loss, and *L* is the length of an infinite fiber used as a QKD link. Under the assumption that the electronic integral time of Alice’s homodyne detector is σt, the excess noise $\zeta_{RB}$ can be rewritten as

(10) $$\zeta_{RB}=\frac{2\Delta_{B}\eta \sigma t}{\eta_{B}T}=\frac{\varpi \left(1-10^{-2\gamma L/10}\right)V_{B}R\sigma t}{\eta_{B}10^{-2\gamma L/10}}.$$

Note that Equation (10) shows the excess noise which is caused by the quantum channel, namely, here $\zeta_{RB}$ can be used to represent ξ.

In the following, we perform an analysis of the effect of phase noise, which commonly exists in a coherent communication system. The excess noise caused by the phase instability is given by

(11) $$\zeta_{p}=\frac{\alpha^{2}\varphi }{N_{0}},$$

where φ represents the phase-noise variance. Here Equation (11) is derived with the assumption of $\alpha^{2}\geq \left(V_{B}+1\right)N_{0}$ [21]. It is worth mentioning that the excess noise φ not only contains the phase noise between the signal and the LO but also the other modulation errors.

On the basis of the above analysis, the overall excess noise outside Alice’s system can be defined as

(12) $$\zeta_{t}=\zeta_{s}+\zeta_{RB}+\zeta_{p}.$$

Note that excess noises $\zeta_{s}$ and $\zeta_{RB}$ are independent of α.

### 3.2. Simulation Results

**Figure 5:**
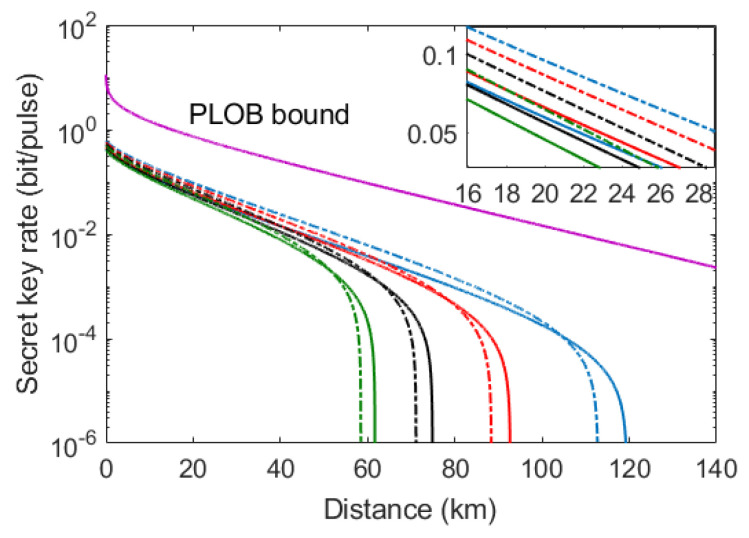
Comparison of secret key rate between the modified protocol (homodyne detection with phase-sensitive amplifier (PSA)) and the original protocol (without PSA) under different imperfect source scenarios. Solid lines represent the original protocol (G = 1) while the dashed lines represent the modified protocol (G = 3). From left to right, the green curves correspond to g=1.015, the black curves correspond to g=1.01, the red curves correspond to g=1.005, and the blue curves correspond to g=1 (no source noise). The simulation parameters are $V_{B}=4$, $\zeta_{p}=10^{-6}$, $\zeta_{RB}=0.02$, η=0.5, $\upsilon_{el}=0.1$.

In Figure 5, we conduct numerical simulations of the asymptotic secret key rate as a function of transmission distance in different imperfect source scenarios. Note that g=1 means no source noise case. We adopt the optimal value of modulation $V_{B}$ in the analysis (see Appendix A). Here, the solid lines in Figure 5 stand for the case of the original protocol (G=1), while the dashed lines represent the case of the modified protocol (a protocol with homodyne detection and a PSA, G = 3). On the one hand, we observed that for each imperfect source scenario, the secret key rate is well improved within a relatively long distance by utilizing an optical preamplifier. On the other hand, we also found that the maximum secure distance of the modified protocol is slightly shorter compared with the original protocol. That is to say, by utilizing the optical amplifier, the secret key rate of the modified protocol increases in a large range of distance with a slight cost of the maximum transmission distance. It is remarkable that the PLOB bound has been plotted in Figure 5, which illustrates the ultimate limit of point-to-point QKD [31]. Here, we should note that the phase noise in our protocol is very small since the LO and signal pulses are generated from the same laser source in the plug-and-play scheme. Therefore, we can achieve the phase noise $\zeta_{p}=10^{-6}$. Detailed calculation of the asymptotic secret key rate is shown in Appendix B.

**Figure 6:**
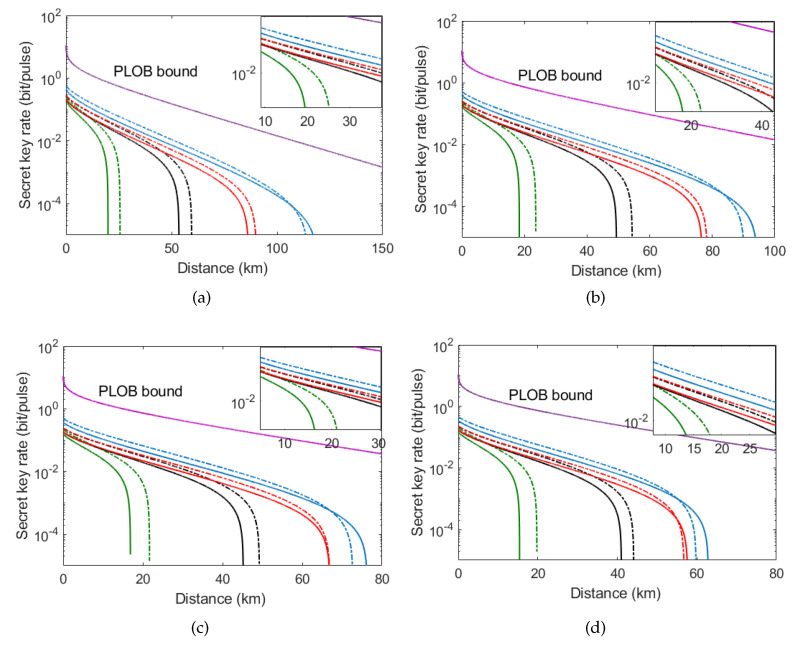
Finite-size secret key rate of SCCQ protocol based on plug-and-play configuration with PSA as a function of transmission distance under different imperfect source scenarios. Solid lines represent the original protocol (G = 1) while the dashed lines represent the modified protocol (G = 3). From left to right, the green curves, the black curves, and the red curves correspond to finite-size scenario of block length $N=10^{6}$, $10^{8}$, and $10^{10}$, respectively, and the blue curves represent the asymptotic scenario. (**a**) The parameter g=1 (no source noise). (**b**) The parameter g=1.005. (**c**) The parameter g=1.01. (**d**) The parameter g=1.015. Other parameters are set to be the same as Figure 5.

In addition, it is necessary to consider the finite-size effect since the length of secret key is impossibly unlimited in practice. Different from the asymptotic case, in the finite-size scenario, the characteristics of the quantum channel cannot be known before the transmission is performed. The reason is that a portion of the exchanged signals needs to be taken advantage of for parameter estimation instead of generating the secret key. We conduct numerical simulations of the finite-size secret key rate in different imperfect source scenarios, as shown in Figure 6. The solid lines in Figure 6 stand for the case of the original protocol (G=1), while the dashed lines represent the case of the modified protocol (G = 3). From left to right, the green curves, the black curves, and the red curves correspond to the finite-size scenario of block length $N=10^{6}$, $10^{8}$, and $10^{10}$, respectively, and the blue curves represent the asymptotic scenario. Here, Figure 6a–d show the proposed protocol with g=1 (no source noise), g=1.005, g=1.01, and g=1.015. We observe that the performance of the asymptotic scenario is better than that of the finite-size scenario whether the PSA is placed at Alice’s detection apparatus or not. Furthermore, the curves of the finite-size scenario are more and more close to the curve of asymptotic case with the increased number of exchanged signals *N*. That is to say that the more exchanged signals we have, the more the signal parameter estimation step can be utilized, and thus the parameter estimation is approaching perfection. Interestingly, for each imperfect source scenario, the finite-size secret key rate of the modified protocol is well improved without the price of reducing the maximum transmission distance, especially for the small-length block, compared with the original protocol, which is different from the asymptotic case. Detailed calculation of finite-size secret key rate is shown in Appendix C.

## 4. Conclusions

We propose a SCCQ protocol based on plug-and-play configuration with an optical amplifier. Benefiting from the plug-and-play scheme where a real local LO is generated from the same laser of quantum signal at Alice’s side, the phase noise existing in our protocol is very small, which can be tolerated by the SCCQ protocol. Therefore, our research may bring the SCCQ technology into real life and thus reduce the cost of QKD effectively. To further improve its capabilities, we inserted an optical amplifier inside Alice’s apparatus. The simulation results show that the secret key rate is greatly enhanced in a large range of distances for each imperfect source scenario in both asymptotic limit and finite-size regime compared with the original protocol.

## Figures and Tables

**Figure A1:**
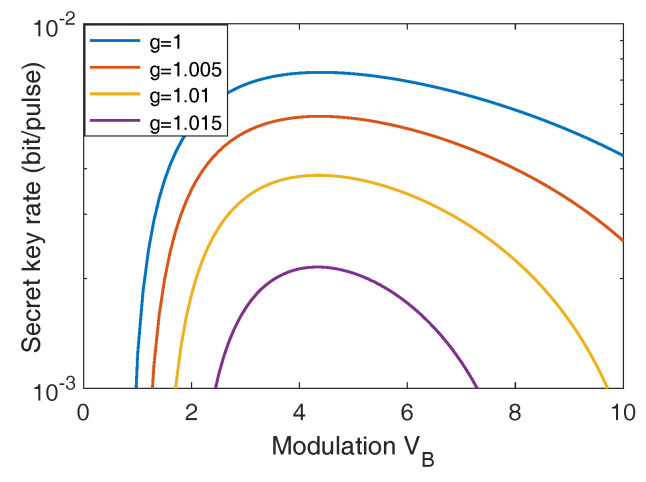
SCCQ protocol based on plug-and-play configuration using homodyne detection with a practical detector, d = 50, G = 1.

**Figure A2:**
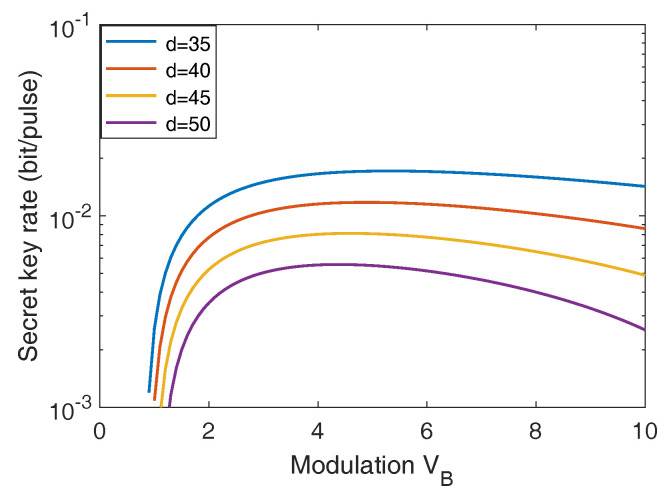
SCCQ protocol based on plug-and-play configuration using homodyne detection with a practical detector, g = 1.005, G = 1.

**Figure A3:**
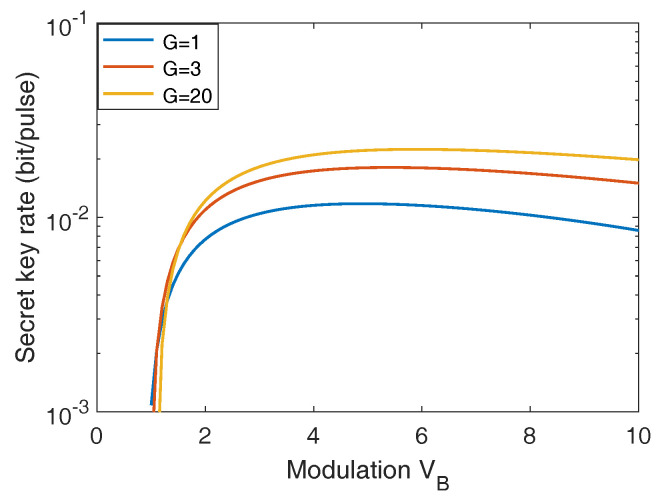
SCCQ protocol based on plug-and-play configuration using homodyne detection with a practical detector, g = 1.005, d = 40.

## Appendix A. Parameter Optimization

To maximize the performance of our protocol, we need to find an optimal Bob’s modulation $V_{B}$. As illustrated in Figure A1, Figure A2 and Figure A3, different values of *g*, *d*, and *G* are, respectively, set to find a public optimal $V_{B}$. From Figure A1, we observe that as the value of parameter *g* increases (the source noise increases), the optimal interval becomes gradually compressed. In addition, the secret key rate also decreases as a result of the increase in parameter *g*. Fortunately, there exists a public interval where we can obtain a public optimal modulation $V_{B}$ for all curves in Figure A1. Namely, we have $V_{B}=4$. Note that in this case, the parameters *d* and *G* are fixed to legitimate values.

The plot of Figure A2 illustrates the relationship between secret key rate and modulation $V_{B}$ under different values of distance *d*. We find that the peak value of secret key rate can be obtained when Bob’s modulation is about 4. In other words, the optimal value of $V_{B}$ in this case is 4.

Furthermore, Figure A3 shows the relationship between secret key rate and modulation $V_{B}$ with different gains of the amplifiers. Here, the optimal value of Bob’s modulation $V_{B}$ is still about 4. That is to say that we achieve the same conclusion (optimal $V_{B}=4$) as above.

In view of above analysis, we can achieve the optimal value of modulation $V_{B}$, namely, $V_{B}=4$, which is deemed as a constant in our protocol.

## Appendix B. Calculation of Asymptotic Secret Key Rate

Here, we calculate the asymptotic secret key rate with reverse reconciliation under the optimal collective attack, which is given by [32]

(A1) $$K_{asym}=fI\left(A:B\right)-\chi_{AE},$$

where *f* is the reconciliation efficiency, I(A:B) represents the Shannon mutual information shared by Alice and Bob, and $\chi_{AE}$ represents the Holevo bound of the information between Eve and Alice.

The mutual information shared by Alice and Bob I(A:B) is expressed as

(A2) $$I\left(A:B\right)=\frac{1}{2}log_{2}\frac{V+\chi_{tot}}{1+\chi_{tot}}.$$

The Holevo bound of the information between Eve and Alice $\chi_{AE}$ can be calculated by [26,27]

(A3) $$\chi_{AE}=\sum_{i=1}^{2}G\left(\frac{\nu_{i}-1}{2}\right)-\sum_{i=3}^{5}G\left(\frac{\nu_{i}-1}{2}\right),$$

where $G\left(x\right)=\left(x+1\right)log_{2}\left(x+1\right)-xlog_{2}x$, and the symplectic eigenvalues $\nu_{1,2,3,4,5}$ are given by

(A4) $$\nu_{1,2}^{2}=\frac{1}{2}\left[A\pm \sqrt{A^{2}-4B}\right],$$

where

(A5) $$A=V^{2}\left(1-2T\right)+2T+T^{2}{\left(V+\chi_{line}\right)}^{2},$$

(A6) $$B=T^{2}{\left(V\chi_{line}+1\right)}^{2},$$

(A7) $$\nu_{3,4}^{2}=\frac{1}{2}\left[C\pm \sqrt{C^{2}-4D}\right],$$

where

(A8) $$C=\frac{A\chi_{hom}+V\sqrt{B}+T\left(V+\chi_{line}\right)}{T(V+\chi_{tot})},$$

(A9) $$D=\frac{\sqrt{B}\left(V+\sqrt{B}\chi_{hom}\right)}{T(V+\chi_{tot})},$$

(A10) $$\nu_{5}=1.$$

Note that in the above equations, $V=V_{B}+1$, the total channel-added noise $\chi_{line}=\frac{1}{T}-1+\zeta_{t}$ and the total noise referred to the channel input $\chi_{tot}=\chi_{line}+\frac{\chi_{hom}}{T}$.

## Appendix C. Secret Key Rate in the Finite-Size Scenario

For the proposed protocol, the finite-size secret key rate is expressed as [33,34]

(A11) $$K_{fini}=\frac{n}{N}\left[fI\left(A:B\right)-\chi_{\epsilon_{PE}}\left(A:E\right)-\Delta \left(n\right)\right],$$

where *f* and I(A:B) are as the same as the aforementioned definitions. Here, *N* stands for the total exchanged signals and *n* stands for the number of signals which is taken advantage of to derive QKD. The remaining signals r=N−n are utilized to estimate the parameter. $\epsilon_{PE}$ represents the failure probability of parameter estimation and $\chi_{\epsilon_{PE}}\left(A:E\right)$ represents the maximum of the Holevo information compatible with the statistics except with probability $\epsilon_{PE}$. Δ(n) is related to the security of the privacy amplification, which is given by

(A12) $$\Delta \left(n\right)=\left(2dimH_{A}+3\right)\sqrt{\frac{log_{2}\left(2/\bar{\epsilon }\right)}{n}}+\frac{2}{n}log_{2}\left(1/\epsilon_{PB}\right),$$

where $\bar{\epsilon }$ and $\epsilon_{PB}$ stand for the smoothing parameter and the failure probability of privacy amplification, respectively. In addition, $H_{A}$ represents the Hilbert space corresponding to the Alice’s raw key. In our protocol, binary bits are taken advantage of to encode the raw key, thus we have $dimH_{A}=2$.

In the finite-size scenario, the covariance matrix $\Gamma_{\epsilon_{PE}}$ needs to be used to calculate $\chi_{\epsilon_{PE}}\left(A:E\right)$. Besides, $\Gamma_{\epsilon_{PE}}$ minimizes the secret key rate with a probability of at least $1-\epsilon_{PE}$. Here, *r* couples of correlated variables ${\left(x_{i},y_{i}\right)}_{i=1\ldots r}$ are sampled to derive the covariance matrix $\Gamma_{\epsilon_{PE}}$. Then, a normal model is used for these correlated variables, which is given by

(A13) y=tx+z,

where $t=\sqrt{T}$ and *z* follow a centered normal distribution with variance $\psi^{2}=1+T\zeta_{t}$. On the basis of Equation (A13), Alice and Bob’s data can be linked. The covariance matrix $\Gamma_{\epsilon_{PE}}$ is given by

(A14) $$\Gamma_{\epsilon_{PE}}=\left(\begin{array}{cc} \left(V_{B}+1\right)I_{2} & t_{min}Z\sigma_{z}\\ t_{min}Z\sigma_{z} & \left(t_{min}^{2}V_{B}+\psi_{max}^{2}\right)I_{2} \end{array}\right),$$

where $t_{min}$ and $\psi_{max}^{2}$ represent the minimum of *t* and maximum of $\psi^{2}$ compatible with sampled couples except with probability $\epsilon_{PE}/2$, and $Z=\sqrt{V_{B}^{2}+2V_{B}}$. Here, we denote the Maximum-likelihood estimators as $\hat{t}$ and ${\hat{\psi }}^{2}$, which can be, respectively, expressed by

(A15) $$\hat{t}=\frac{\sum_{i=1}^{r}x_{i}y_{i}}{\sum_{i=1}^{r}x_{i}^{2}}\;and\;{\hat{\psi }}^{2}=\frac{1}{r}\sum_{i=1}^{r}{\left(y_{i}-\hat{t}x_{i}\right)}^{2}.$$

According to this, $t_{min}$ (the minimum of *t*) and $\psi_{max}^{2}$ (the maximum of $\psi^{2}$) can be derived by

(A16) $$\begin{array}{cc} t_{min} & \approx \hat{t}-z_{\epsilon_{PE}/2}\sqrt{\frac{{\hat{\psi }}^{2}}{rV_{B}}},\\ \psi_{max}^{2} & \approx {\hat{\psi }}^{2}+z_{\epsilon_{PE}/2}\frac{\sqrt{2}{\hat{\psi }}^{2}}{\sqrt{r}}, \end{array}$$

where $z_{\epsilon_{PE}/2}$ is such that $1-erf(z_{\epsilon_{PE}/\sqrt{2}})/2=\epsilon /2$, and $erf\left(x\right)=\frac{2}{\sqrt{\pi }}\int_{0}^{x}e^{-t^{2}}dt$ stands for error function. In order to theoretically analyze our protocol, the expected values of $\hat{t}$ and ${\hat{\psi }}^{2}$ are, respectively, given by

(A17) $$\begin{array}{cc} E[\hat{t}] & =\sqrt{T},\\ E[{\hat{\psi }}^{2}] & =1+T\zeta_{t}. \end{array}$$

Then, we can calculate $t_{min}$ and $\psi_{max}^{2}$ as follows:

(A18) $$\begin{array}{cc} t_{min} & \approx \sqrt{T}-z_{\epsilon_{PE}/2}\sqrt{\frac{1+T\zeta_{t}}{rV_{B}}},\\ \psi_{max}^{2} & \approx 1+T\zeta_{t}+z_{\epsilon_{PE}/2}\frac{\sqrt{2}\left(1+T\zeta_{t}\right)}{\sqrt{r}}. \end{array}$$

The optimal value for the error probabilities can be taken as being

(A19) $$\bar{\epsilon }=\epsilon_{PE}=\epsilon_{PB}=10^{-10}.$$

Then, the secret key rate in the finite-size scenario can be calculated by taking advantage of the derived bounds $t_{min}$ and $\psi_{max}^{2}$.
